# Comparison of olanexidine versus povidone‐iodine as a preoperative antiseptic for reducing surgical site infection in both scheduled and emergency gastrointestinal surgeries: A single‐center randomized clinical trial

**DOI:** 10.1002/ags3.12675

**Published:** 2023-04-16

**Authors:** Akira Umemura, Akira Sasaki, Hisataka Fujiwara, Kazuho Harada, Satoshi Amano, Naoto Takahashi, Yota Tanahashi, Takayuki Suto

**Affiliations:** ^1^ Department of Surgery Iwate Medical University School of Medicine 2‐1‐1 Idaidori, Yahaba Japan; ^2^ Department of Surgery Morioka Municipal Hospital 5‐15‐1 Motomiya Morioka Japan; ^3^ Department of Anesthesiology Morioka Municipal Hospital 5‐15‐1 Motomiya Morioka Japan

**Keywords:** antiseptic, olanexidine, povidone‐iodine, randomized clinical trial, surgical site infection

## Abstract

**Aim:**

Surgical site infection (SSI) is one of the most common postoperative complications in gastrointestinal surgery. To clarify the superiority of 1.5% olanexidine, we conducted a randomized prospective clinical trial that enrolled patients undergoing gastrointestinal surgery with operative wound classes II–IV.

**Methods:**

To evaluate the efficacy of 1.5% olanexidine in preventing SSIs relative to 10% povidone‐iodine, we enrolled 298 patients in each group. The primary outcome was a 30‐day SSI, and the secondary outcomes were incidences of superficial and deep incisional SSI and organ/space SSI. In addition, subgroup analyses were performed.

**Results:**

The primary outcome of the overall 30‐day SSI occurred in 38 cases (12.8%) in the 1.5% olanexidine group and in 53 cases (18.0%) in the 10% povidone‐iodine group (adjusted risk ratio: 0.716, 95% confidence interval: 0.495–1.057, *p* = 0.083). Organ/space SSI occurred in 18 cases (6.1%) in the 1.5% olanexidine group and in 31 cases (10.5%) in the 10% povidone‐iodine group, with a significant difference (adjusted risk ratio: 0.587, 95% confidence interval: 0.336–0.992, *p* = 0.049). Subgroup analyses revealed that SSI incidences were comparable in scheduled surgery (relative risk: 0.809, 95% confidence interval: 0.522–1.254) and operative wound class II (relative risk: 0.756, 95% confidence interval: 0.494–1.449) in 1.5% olanexidine group.

**Conclusion:**

Our study revealed that 1.5% olanexidine reduced the 30‐day overall SSI; however, the result was not significant. Organ/space SSI significantly decreased in the 1.5% olanexidine group. Our results indicate that 1.5% olanexidine has the potential to prevent SSI on behalf of povidone‐iodine.

## Graphical abstract

This study demonstrated 1.5% olanexidine reduced the overall SSI incidence relative to 10% povidone‐iodine (*p* = 0.083), and significantly reduced organ/space SSI (*p* = 0.049). Subgroup analyses revealed that emergency surgery and operative wound class did not increase SSI incidence.

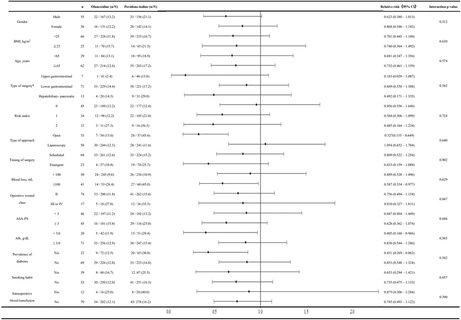

## 1 INTRODUCTION

In gastrointestinal surgery, surgical site infection (SSI) is one of the most common postoperative complications.^1^, ^2^, ^3^ In general, SSI prolongs hospitalization and increases medical costs for wound care, antibiotics, and interventions. The 2021 annual report of Japan Nosocomial Infections Surveillance (JANIS) showed that SSI incidence rates after upper gastrointestinal, colorectal, and hepatobiliary‐pancreatic surgeries were 6.0%–16.7%, 8.5%–10.4%, and 6.3%–22.7%, respectively.^4^ Multiple management protocols have been implemented to prevent SSI; among these, appropriate prophylactic antibiotics and preoperative skin antiseptics are the most important to minimize the risk of SSI.^1^, ^2^, ^3^

In current clinical practice across the world, povidone‐iodine and chlorhexidine are widely used as preoperative antiseptics. Furthermore, povidone‐iodine has been popularly used in daily clinical practice for more than 50 years. In contrast, some clinical trials have demonstrated that the application of chlorhexidine to patients before clean or clean–contaminated surgery is associated with lower SSI incidence relative to patients with povidone‐iodine antisepsis.^5^, ^6^ Based on this evidence, chlorhexidine is the most widely used antiseptic in the United States and Europe. However, the quality of these studies is not sufficient to provide a strong recommendation for the choice of preoperative antiseptics.

Recently, 1.5% olanexidine (Olanedine; Otsuka Pharmatceutical Factory, Tokushima, Japan) has been commercially available as a preoperative antiseptic in Japan. In addition, some prospective and retrospective comparative studies have been conducted on gastrointestinal surgery.^7^, ^8^, ^9^, ^10^, ^11^ However, the indications of all studies are limited to clean–contaminated (operative wound class II) gastrointestinal surgery. To clarify the true superiority of 1.5% olanexidine, we conducted a randomized prospective clinical trial to enroll patients undergoing gastrointestinal surgery with operative wound classes II–IV, including emergency surgery. Using the abovementioned clinical trials, the present study assessed the SSI‐preventing effects of 1.5% olanexidine compared with 10% povidone‐iodine.

## 2 METHODS

### 2.1 Study design

We conducted a single‐center, prospective, randomized, open‐endpoint trial to evaluate the efficacy of 1.5% olanexidine in preventing SSIs compared with the 10% povidone‐iodine group in gastrointestinal surgery. This clinical trial was approved by the institutional ethics committee of Morioka Municipal Hospital (H30‐1), here in accordance with the Declaration of Helsinki and the National Clinical Trials Registry (UMIN000033830) before patient recruitment. As a prospective randomized controlled trial, the study strategy was constructed following the CONSORT 2010 statement.^12^

### 2.2 Eligibility criteria

Eligible participants were aged 20 years or above and underwent either classes II–IV scheduled or emergency gastrointestinal, hepatobiliary, or pancreatic surgery. In the present study, surgical procedures with intestinal resections were defined as eligible surgical procedures; therefore, cholecystectomy, splenectomy, and hepatectomy without intestinal resections were excluded. In contrast, appendectomy, perforated peritonitis, and other emergency surgical procedures with intestinal resection were also included as class III or IV surgeries. All patients provided written informed consent before randomization.

Exclusion criteria were as follows: (1) allergy to olanexidine gluconate or povidone‐iodine, (2) antimicrobial therapy the day before surgery, and (3) patients who were deemed by surgeons to be inappropriate for participation.

### 2.3 Randomization and sample size setting

We employed three adjustment factors for randomization: age, gender, and type of surgery. Both olanexidine and povidone‐iodine were recognized by color after antisepsis; therefore, blinding was not employed.

Regarding the sample size, an enrollment ratio was set at 1:1, and 298 patients for each group were required to follow the backgrounds and calculations. The average SSI incidence rate for gastrointestinal surgery was 3.7%–22.7% (average 10.9%), according to the 2021 SSI open report of JANIS.^4^ Previous reports revealed that the SSI incidence of contaminated and infected abdominal surgery was about 15%–20%^13^, ^14^; hence, the SSI incidence rate for povidone‐iodine was set as 18% by including contaminated and infected surgeries. We assumed that olanexidine can reduce the SSI incidence rate to 10%, with a power of 80% and an alpha error of 5%.

### 2.4 Study protocol

A preoperative culture of the umbilicus was routinely performed after final preoperative bathing. Culture sampling was performed after bed bathing the patients who required an emergency operation. Following the induction of general anesthesia, hair removal was performed using a clipper, if necessary. Surgical skin antisepsis with 1.5% olanexidine or 10% povidone‐iodine was then administered to cover the entire surgical site. In the present study, the entire surgical site was defined as the area from the level of the papilla to the upper thighs. Antiseptics were allowed to dry for 3 min, and we then began to perform the surgery.

We used other procedures to prevent SSI along with bundles as follows: (1) Antibiotic prophylaxis was administered within 60 min before incision via intravenous dripping. A repeated dose of antibiotic prophylaxis was administered 3 h after starting the surgery. We used cefazoline for upper gastrointestinal surgery, cefmetazole for colorectal surgery, and a combination of cefoperazone and sulbactam for hepatobiliary and pancreatic surgery. (2) We routinely employed the double gloves technique and changed gloves before closure. (3) We routinely used plastic double‐ring wound protectors in both open surgery and small incisions for laparoscopic procedures. (4) We used new surgical suturing instruments for wound closure. (5) Triclosan antibacterial monofilament sutures were employed for wound closure. (6) Intraoperative normothermia was maintained by continuously monitoring body temperature and warming devices.

After abdominal wall closure, we checked the intraoperative culture of the wound to evaluate the causative bacteria of the SSIs. Wound irrigation was performed with 500 mL of sterilized normal saline before skin closure, and skin closure was routinely performed using absorbable monofilament buried sutures for laparoscopic surgery and a barbed suture device for open surgery.

### 2.5 Evaluation of SSI

Regarding usual wound checking, responsible surgeons examined the presence or absence of SSI daily during the hospital stay and at every outpatient visit until 30 days after surgery. The wound condition was diagnosed as superficial SSI when it satisfied all the following criteria: (1) infection must be confirmed within 30 days after surgery; (2) the extent of the infection is limited within the skin and subcutaneous tissue; and (3) organisms were isolated from an aseptically obtained culture of fluid or tissue from the superficial incision, and purulent discharge (with or without laboratory confirmation), signs of infection (pain, tenderness, localized swelling, redness, and heat), and a superficial incision, which was deliberately opened by the surgeon unless the incision was culture‐negative, were observed.

In contrast, organ/space SSI was also diagnosed by the following criteria: (1) infection should be confirmed within 30 days after surgery, (2) organisms were isolated from an aseptically obtained culture of fluid or tissue from the organ/space infection site, and (3) the infection site, including the abscess cavity, must be detected in the operative area by multimodal diagnostic tools. When organ/space SSI and other wound complications were found in the same patient, we counted as organ/space SSI; therefore, duplicated count of every SSI incidence was strictly avoided.

### 2.6 Outcome and data collection

The primary outcome was a 30‐day SSI after surgery, here following the Centers of Disease Control and Prevention guidelines.^15^ In the present study, incidences of superficial and deep incisional SSI and organ/space SSI were also analyzed as secondary outcomes.

We preoperatively collected the following laboratory data: serum albumin and white blood cell (WBC), a fraction of neutrophil, lymphocyte, and platelet counts. In addition, patient background, body mass index (BMI), prevalence of diabetes, smoking habit, physical status approved by the American Society of Anesthesiologists (ASA‐PS),^16^ timing of surgery (scheduled or emergent), types of surgery (upper gastrointestinal, lower gastrointestinal, hepatobiliary‐pancreatic, and overlapping), types of approach (open or laparoscopy), risk index (0–3) approved by JANIS,^4^ and operative wound class (II, III, and IV) were collected. Operative parameters, operating time, amount of blood loss, administration of intraoperative blood transfusion, and duration of hospital stay were also measured.

We performed wound cultures in all enrolled cases for a maximum of three times: umbilical skin culture before surgery, wound culture before skin closure during surgery, and SSI diagnosis.

### 2.7 Statistical analysis

All data are presented as numbers for categorical variables and as means ± SD for continuous variables. Statistical analysis was performed using chi‐square tests for categorical variables and Student's t‐tests or Mann–Whitney U tests for continuous variables. We used paired t‐tests and Wilcoxon tests for continuous variables to enable a comparison of all parameters between pre‐ and postoperative measures.

The primary outcome was analyzed with Fisher's exact test, and the Mantel–Haenszel test was also employed to adjust for the types of surgery. We also performed subgroup analyses using stratified Mantel–Haenszel tests to investigate the risk factors of SSI incidence. The stratified parameters for subgroup analyses were as follows: gender (male vs. female), BMI (<25 kg/m^2^ vs. ≥25 kg/m^2^), age (younger than 5 years vs. 65 years or older), types of surgery (upper gastrointestinal, lower gastrointestinal, and hepatobiliary‐pancreatic), risk index (0, 1, and 2), types of approach (open vs. laparoscopy), the timing of surgery (scheduled surgery vs. emergency surgery), intraoperative blood loss (<100 mL vs. ≥100 mL), operative wound class (II vs. III and IV), ASA‐PS (1 and 2 vs. 3 and 4), preoperative serum albumin level (<3.0 g/dL vs. ≥3.0 g/dL), prevalence of diabetes (yes vs. no), smoking habit (yes vs. no), and administration of intraoperative blood transfusion (yes vs. no).

All *p* values less than 0.05 using two‐sided analyses were considered significant. All statistical analyses were performed using JMP statistical software version 15 (SAS Institute).

## 3 RESULTS

### 3.1 Patient flowchart and characteristics

**FIGURE 1:**
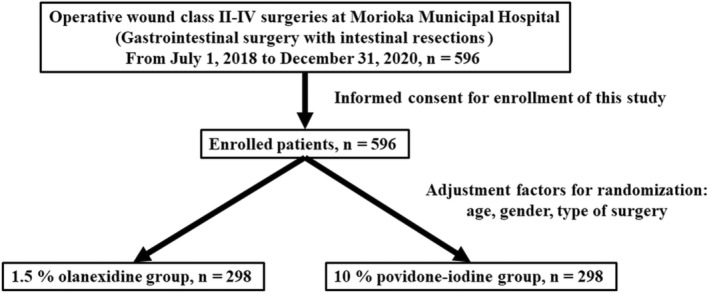
Patients flowchart of this study.

Between July 1, 2018, and December 31, 2020, we enrolled 596 patients at Morioka Municipal Hospital, and 298 patients were assigned to the 1.5% olanexidine group and 298 to the 10% povidone‐iodine group. The patient flowchart of this study is shown in Figure 1. We excluded patients who did not meet the inclusion criteria; patients with an ASA‐PS of 5 or a history of iodine allergy before enrollment were also excluded. Therefore, 298 patients in each group were included in the full‐analysis set. We then used an intention‐to‐treat analysis.

Table 1 shows the patient characteristics and operative outcomes of each group. No significant differences were observed in the adjusting factors, prevalence of diabetes, serum albumin, neutrophil count, lymphocyte count, platelet count, or ASA‐PS. By contrast, significant differences were observed in WBC (7162.6 WBC/μL vs. 7898.8 WBC/μL, *p* = 0.025), operative wound class (*p* = 0.048), operating time (*p* = 0.026), and timing of emergency surgery (12.4% vs. 24.8%).

**TABLE 1:** Baseline characteristics and operative outcomes.

	All patients (*n* = 596)	Olanexidine (*n* = 298)	Povidone‐iodine (*n* = 298)	*p* Value
Age, years	69.3 ± 15.9	69.1 ± 15.0	69.5 ± 16.8	0.740
Gender				
Male, *n* (%)	323 (54.2)	167 (56.0)	156 (52.3)	0.366
Female, *n* (%)	273 (45.8)	131 (44.0)	142 (47.7)	
BMI, kg/m^2^	22.3 ± 4.2	22.3 ± 3.9	22.4 ± 4.4	0.748
Prevalence of diabetes, *n* (%)	137 (23.0)	72 (24.2)	65 (21.8)	0.539
Smoking habit				
Yes, *n* (%)	95 (15.9)	48 (16.0)	47 (15.8)	0.911
No, *n* (%)	501 (84.1)	250 (84.0)	251 (84.2)	
Serum albumin, g/dL	3.8 ± 0.7	3.8 ± 0.7	3.7 ± 0.7	0.526
WBC, /μL	7528.8 ± 3997.8	7162.6 ± 3435.5	7898.8 ± 4470.5	0.025^a^
Neutrophile count, /μL	5392.2 ± 3836.1	5104.1 ± 3435.5	5669.4 ± 4122.8	0.091
Lymphocyte count, /μL	1550.1 ± 1004.0	1552.1 ± 895.7	1548.2 ± 1099.8	0.965
Platelet count, ×10^4^/μL	26.7 ± 10.9	26.7 ± 10.9	26.6 ± 11.0	0.930
ASA‐PS, *n* (%)				
1	110 (18.5)	58 (19.5)	52 (17.4)	0.235
2	269 (45.1)	139 (46.6)	130 (43.6)	
3	198 (33.2)	96 (32.2)	102 (34.3)	
4	19 (3.2)	5 (1.7)	14 (4.7)	
Risk index, *n* (%)				
0	366 (61.4)	189 (63.3)	177 (59.4)	0.413
1	203 (34.1)	98 (33.0)	105 (35.2)	
2	27 (4.5)	11 (3.7)	16 (5.4)	
Timing of surgery, *n* (%)				
Scheduled	485 (81.4)	261 (87.5)	224 (75.2)	< 0.001^a^
Emergent	111 (18.6)	37 (12.4)	74 (24.8)	
Type of surgery, *n* (%)				
Upper gastrointestinal	87 (14.6)	41 (13.7)	46 (15.4)	0.613
Lower gastrointestinal	438 (73.5)	221 (74.2)	217 (72.8)	
Hepatobiliary‐pancreatic	59 (9.9)	28 (9.4)	31 (10.4)	
Overlapping	12 (2.0)	8 (2.7)	4 (1.4)	
Type of approach, *n* (%)				
Open	111 (18.6)	54 (18.1)	57 (19.1)	0.281
Laparoscopy	485 (81.4)	244 (81.9)	241 (80.9)	
Operative wound class, *n* (%)				
II	542 (91.0)	280 (94.0)	262 (88.0)	0.048^a^
III	24 (3.9)	8 (2.7)	16 (5.3)	
IV	30 (5.1)	10 (3.3)	20 (6.7)	
Operating time, min	158.8 ± 80.9	166.2 ± 79.6	151.4 ± 81.7	0.026^a^
Blood loss, mL	83.8 ± 196.7	77.4 ± 183.0	90.3 ± 209.9	0.427
Intraoperative blood transfusion				
Yes, *n* (%)	36 (6.0)	16 (5.4)	20 (6.7)	0.491
No, *n* (%)	560 (94.0)	282 (94.6)	278 (93.3)	
Postoperative hospital stay, days	10.5 ± 9.9	10.0 ± 9.5	11.0 ± 10.2	0.256

*Note*: Values are the mean ± SD.

Abbreviations: ASA‐PS, physical status approved by the American Society of Anesthesiologists; WBC, white cell count.

^a^ Parameters with *p* < 0.05.

### 3.2 Primary and secondary outcomes

The incidences of SSIs in each group and the statistical results are shown in Table 2. The primary outcome of the overall 30‐day SSI assessed in the full‐analysis set occurred in 38 cases (12.8%) in the 1.5% olanexidine group and in 53 cases (18.0%) in the 10% povidone‐iodine group (odds ratio: 0.676, 95% confidence interval: 0.431–1.059, adjusted risk ratio: 0.716, 95% confidence interval: 0.495–1.057, *p* = 0.083).

**TABLE 2:** The results of primary outcome.

	Olanexidine (*n* = 298)	Povidone‐iodine (*n* = 298)	Odds ratio (95% confident interval)	Adjusted risk ratio (95% confident interval)	*p* Value
30‐day SSI, *n* (%)	38 (12.8)	53 (18.0)	0.676 (0.431–1.059)	0.716 (0.495–1.057)	0.083
Incisional, *n* (%)	20 (6.7)	22 (7.5)	0.857 (0.460–1.596)	0.925 (0.643–1.228)	0.630
Organ/space, *n* (%)	18 (6.1)	31 (10.5)	0.547 (0.301–0.997)	0.587 (0.336–0.992)	0.049^a^

Abbreviation: SSI; surgical site infection.

*Note*: Values are the mean ± SD.

^a^ Parameters with *p* < 0.05.

No significant difference was found in the incidence of superficial incisional SSI (6.7% vs. 7.5%, odds ratio: 0.857, 95% confidence interval: 0.460–1.596, adjusted risk ratio: 0.925, 95% confidence interval: 0.643–1.228, *p* = 0.630). In the present study, deep incisional SSI was not found in either group. Organ/space SSI occurred in 18 cases (6.1%) in the 1.5% olanexidine group and in 31 cases (10.5%) in the 10% povidone‐iodine group, here showing a significant difference in organ/space SSI incidence (odds ratio: 1.813, 95% confidence interval: 1.015–3.324, adjusted risk ratio: 0.587, 95% confidence interval: 0.336–0.992, *p* = 0.049).

### 3.3 Subgroup analyses

**FIGURE 2:**
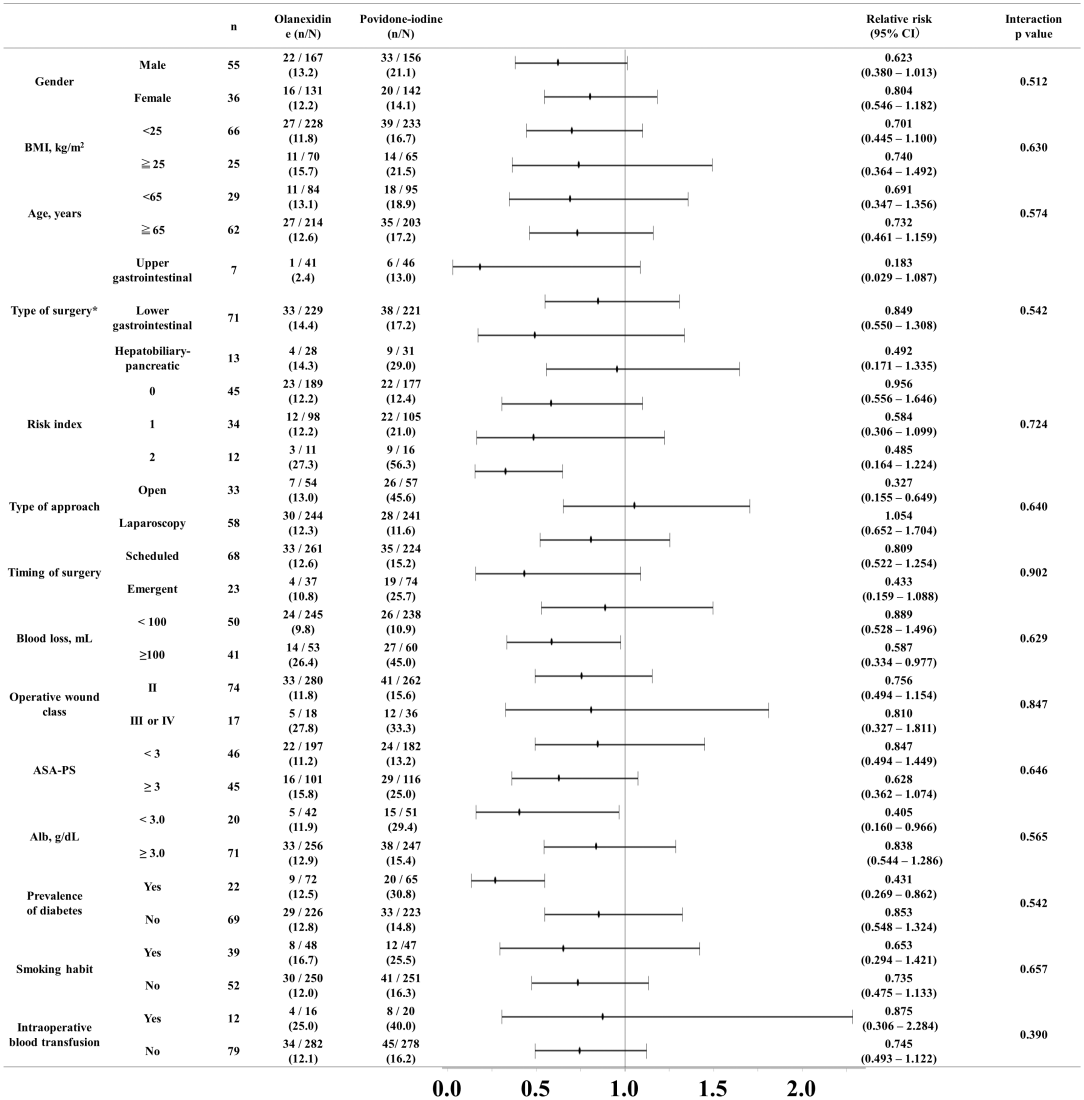
Subgroup analyses of overall SSI in the full‐analysis set. Abbreviation: BMI; body mass index, ASA‐PS; physical status approved by the American Society of Anesthesiologists, CI, confident interval. *All patients of overlapping surgeries underwent lower gastrointestinal surgeries; therefore, these patients were stratified in the lower gastrointestinal group in this analyses.

The results of the subgroup analyses are shown in Figure 2. We analyzed whether SSI incidence was associated with both the type of antisepsis and other background characteristics. The overall SSI rate was low in the 1.5% olanexidine group. The incidence of SSI was significantly low in patients who underwent open surgery in the 1.5% olanexidine group (13.0% vs. 45.6%, relative risk: 0.327, 95% confidence interval: 0.155–0.649). Higher intraoperative blood loss (26.4% vs. 45.0%, relative risk: 0.587, 95% confidence interval: 0.334–0.977), lower serum albumin levels (11.9% vs. 29.4%, relative risk: 0.405, 95% confidence interval: 0.160–0.966) and the prevalence of diabetes (12.5% vs. 30.8%, relative risk: 0.431, 95% confidence interval: 0.269–0.862) were also associated with higher SSI incidence in the 10% povidone‐iodine group. The incidence of SSI in patients who underwent scheduled surgery was comparable in 1.5% olanexidine group (12.6% vs. 15.2%, relative risk: 0.809, 95% confidence interval: 0.522–1.254). Furthermore, overall SSI rate was comparable in patients with operative wound class II in 1.5% olanexidine group (11.8% vs. 15.6%, relative risk: 0.756, 95% confidence interval: 0.494–1.449).

### 3.4 Comparison between SSI‐causing bacteria and preoperative wound cultures

A positive bacterial culture of SSI was observed in 20 patients (52.6%) in the 1.5% olanexidine group and in 19 patients (39.6%) in the 10% povidone‐iodine group. Table 3 shows the concordance rate between SSI‐causing organisms and preoperative or wound cultures in the 1.5% olanedexidine group. Preoperative umbilical culture revealed that most cultured bacteria were coagulase‐negative *Staphylococcus* sp., *Corynebacterium* sp., and *Micrococcus* sp. However, some bacteria in the gut microbiota, such as *Enterobacter aerogenes*, *Enterococcus faecalis*, *Escherichia coli*, *Pseudomonas aeruginosa*, and *Serratia marcescens*, were found. A positive wound culture just before skin closure was observed in 22 patients (57.9%) in the 1.5% olanexidine group. The breakdown of the cultured bacteria was as follows: β‐*Streptococcus* sp. in two patients, coagulase negative *Staphylococcus* sp. in seven patients, *Enterococcus faecalis* in three patients, *Enterococcus* sp. in three patients, *Enterobacter cloacae* in two patients, *Pseudomonas aeruginosa* in two patients, and other bacteria in one to two patients. Regarding SSI‐causing bacteria, *Enterococcus faecalis* and *Enterococcus* sp. were the dominant bacteria. Furthermore, 80.0% of these bacteria continued to infect after surgery. *Enterobacter cloacae*, methicillin‐resistant *Staphylococcus aureus*, and *Pseudomonas aeruginosa* were detected as persistent infection‐type bacteria, despite using 1.5% olanexidine. Table 4 also shows the concordance rate between SSI‐causing organisms and preoperative or wound cultures in the 10% povidone‐iodine group. Preoperative umbilical culture revealed that some bacteria in the gut microbiota were similar to those found in the 1.5% olanexidine group. A positive wound culture just before skin closure was observed in 34 cases (58.6%) of the 10% povidone‐iodine group. The culture‐positive SSI rate in the 10% povidone‐iodine group was 55.2%, and no significant difference was found compared with what was found that in the 1.5% olanexidine group (47.8%, *p* = 0.291). Regarding SSI‐causing bacteria, *Enterococcus faecalis* and *Escherichia coli* were the dominant bacteria in the gut microbiota. In contrast, coagulase‐negative *Staphylococcus sp*. and *Corynebacterium* sp. were also detected more frequently in the 10% povidone‐iodine group compared with the 1.5% olanexidine group. In the present study, 10% povidone‐iodine could not maintain its antiseptic effects during surgery because a high concordance rate was observed in some SSI‐causing bacteria.

**TABLE 3:** Concordance rate between SSI‐causative organisms and preoperative or wound cultures in 1.5% olanexidine group.

	Preoperative	Wound	SSI	Concordance rate preoperative/SSI, *n* (%) wound/SSI, *n* (%)
Negative	2	16	18	
*Acinetobacter* sp.	1	0	0	
*Bacteroides* sp.	0	0	2	
*β‐Streptococcus* sp.	0	2	0	
Coagulase negative *Staphylococcus* sp.	27	7	1	1/1 (100.0)
				0/0 (0.0)
*Corynebacterium* sp.	20	0	2	2/2 (100.0)
				0/2 (0.0)
*γ‐Streptococcus*. sp.	0	0	1	
*Enterobacter aerogenes*	1	0	1	
*Enterobacter cloacae*	0	2	3	0/0 (0.0)
				2/2 (100.0)
*Enterococcus faecalis*	1	3	8	0/0, 0.0
				2/3, 66.7
*Enterococcus* sp.	0	3	5	0/0 (0.0)
				2/3 (66.7)
*Escherichia coli*	2	1	3	
*Klebsiella oxytoca*	0	1	0	
*Klebsiella pneumoniae*	0	1	0	
Methicillin‐sensitive *Staphylococcus aureus*	1	1	0	
Methicillin‐resistant *Staphylococcus aureus*	1	1	1	0/1 (0.0)
				1/1 (100.0)
*Micrococcus* sp.	1	0	0	
*Morganella morganii*	0	0	1	
*Peptostreptococcus* sp.	1	0	0	
*Porphyromonas* sp.	1	0	0	
*Proteus mirabilis*	1	0	0	
*Pseudomonas aeruginosa*	1	2	4	0/1 (0.0)
				1/2 (50.0)
*Serratia marcescens*	1	0	0	
*Yeast‐like fungus*	1	0	0	

*Note*: Duplicated cultured bacteria in the same patients are counted separately.

Abbreviation: SSI, surgical site infection.

**TABLE 4:** Concordance rate between SSI‐causative organisms and preoperative or wound cultures in 10% povidone‐iodine group.

	Preoperative	Wound	SSI	Concordance rate preoperative/SSI, *n* (%) wound/SSI, *n* (%)
Negative	4	24	32	
*Acinetobacter* sp.	1	0	1	
*Alcaligenes* sp.	1	0	0	
*α‐Streptococcus* sp.	0	1	0	
*Bacteroides fragilis*	0	0	1	
*Bacteroides* sp.	0	0	1	
*β‐Streptococcus* sp.	0	1	0	
*Citrobacter freundii*	0	1	0	
*Citrobacter koseri*	0	1	0	
*Citrobacter* sp.	2	1	0	
*Clostridium* sp.	0	0	0	
Coagulase negative *Staphylococcus* sp.	39	5	4	2/4 (50.0)
				2/4 (50.0)
*Corynebacterium* sp.	28	20	4	3/4 (75.0)
				0/4 (0.0)
*γ‐Streptococcus*. sp.	0	0	1	
*Enterobacter cloacae*	0	1	3	0/0 (0.0)
				1/1 (100.0)
*Enterobacter* sp.	0	1	0	
*Enterococcus faecalis*	1	8	7	0/1 (0.0)
				7/8 (87.5)
*Enterococcus* sp.	1	2	1	0/1 (0.0)
				1/2 (50.0)
*Escherichia coli*	2	9	5	2/2 (100.0)
				5/5 (100.0)
*Klebsiella oxytoca*	0	3	0	
*Klebsiella pneumoniae*	1	3	3	0/1 (0.0)
				1/3 (33.3)
Methicillin‐ sensitive *Staphyrococcus aureus*	1	0	0	
*Morganella morganii*	1	1	1	
*Proteus mirabilis*	0	0	1	
*Proteus vulgaris*	0	1	0	
*Proteus* sp.	1	0	0	
*Pseudomonas aeruginosa*	5	1	2	1/2 (50.0)
				0/1 (0.0)
*Serratia marcescens*	1	0	0	
*Stenotrophomonas maltophilia*	1	0	0	
*Yeast‐like fungus*	1	4	2	1/1 (100.0)
				2/2 (50.0)

*Note*: Duplicated cultured bacteria in the same patients are counted separately.

Abbreviation: SSI, surgical site infection.

## 4 DISCUSSION

The present randomized controlled trial presents two major results. First, 1.5% olanexidine reduced the overall SSI incidence relative to 10% povidone‐iodine as a preoperative antisepsis; however, the result was not significant (*p* = 0.083). Second, 1.5% olanexidine significantly (*p* = 0.049) reduced organ/space SSI. In studies that have clarified the efficacy of antisepsis, the timing of surgery and operative wound class were considered to exclude selection biases; however, homogenization in clinical trials diverges from clinical practice. To compensate for this discrepancy, we enrolled patients undergoing emergency surgery or/with higher operative wound class (contaminated and infected) for the present study. Subgroup analyses revealed that emergency surgery and operative wound class did not increase SSI incidence. The present study was the first single‐center randomized prospective trial to investigate the efficacy of 1.5% olanexidine for SSI prevention relative to 10% povidone‐iodine in patients undergoing clean–contaminated (class II), contaminated (class III), and infected (class IV) surgeries.

The bactericidal mechanism of olanexidine has a higher affinity for bacterial surface proteins, such as the lipoteichoic acid of Gram‐positive bacteria and lipopolysaccharide of Gram‐negative bacteria, when compared with chlorhexidine.^17^ In addition, olanexidine has a stronger disruptive effect on the bacterial membrane compared with chlorhexidine; therefore, as a bactericidal effect, the bacterial cell wall ruptures, causing irreversible damage and leakage of intracellular components.^17^ In contrast, olanexidine also has an inhibitory action on inflammatory chemokines. Nii et al. reported that 0.1% olanexidine significantly inhibits the production of inflammatory chemokines, such as interleukin 8, C‐C motif ligand 20, and growth‐related oncogene protein‐α, which are stimulated by *Porphyromonas gingivalis* lipopolysaccharide in oral epithelial cells in in vitro experiments.^18^ Furthermore, olanexidine has an immediate disinfectant effect on a wide range of bacteria within 30 s and substantivity until 6 h relative to chlorhexidine and 10% povidone‐iodine.^19^ Olanexidine is a newly invented antiseptic agent approved in Japan in 2015. Since then, it has been used as a routine preoperative antiseptic in clinical practice based on prior basic studies.

To investigate the clinical efficacy of olanexidine as an antiseptic agent, Asukai et al. first reported a retrospective study in the field of orthopaedic surgery, with SSI incidence as the primary outcome.^20^ However, this study could not demonstrate the superiority of 1.5% olanexidine compared to the 10% povidone‐iodine group (1.80% vs. 2.38%, *p* = 0.500).^20^ Orthopaedic surgery requires operative wound class I surgery because osteomyelitis and device‐associated infection should be avoided; therefore, the usual SSI incidence is lower in orthopaedic surgery than in gastrointestinal surgery, and a significant difference in clean orthopaedic surgery is difficult to identify. Table 5 shows a summary of previous studies regarding olanexidine in gastrointestinal surgeries. In 2020, Obara et al. conducted a multicenter, randomized, controlled trial in clean–contaminated (class II) gastrointestinal and hepatobiliary‐pancreatic surgeries and found a significant difference between 1.5% olanxidine and 10% povidone‐iodine.^7^ In 2022, Kubo et al. also reported a single‐center retrospective study of stomach and colon cancer surgeries and found that 1.5% olanexidine is superior in the prevention of overall (2.7% vs. 10.3%, *p* = 0.020) and superficial incisional SSI (2.2% vs. 8.6%, *p* = 0.034) incidences compared with that in 10% povidone‐iodine.^11^ In contrast, two negative propensity score matching studies regarding olanexidine have been conducted; Kojima et al. found no significant difference in SSI incidence in patients undergoing colorectal surgery compared with to 10% povidone‐iodine (8.6% vs. 8.6%, *p* = 1.000).^9^ Fujita et al. first reported a comparative study using propensity score matching analysis between 1.5% olanexidine and 1.0% chlorhexidine in thoracic esophagectomy; however, the study could not demonstrate any significant differences in SSI incidences.^10^ This study could not demonstrate any significant decrease in 30‐day SSI incidence; however, a significant reduction in organ/space SSI incidence was observed (*p* = 0.049).

**TABLE 5:** Reported comparative studies of olanexidine for gastrointestinal surgeries.

Author	Year	Study design	Targeted surgery	Reference arm	Primary outcome	Secondary outcomes
Obara H, et al.^7^	2020	Multicenter	Operative wound class II gastrointestinal and hepatobiliary‐pancreatic surgery	10% povidone‐iodine	30‐day SSI	Superficial incisional SSI 1% vs. 4%, *p* = 0.03^a^
		Randomized controlled trial			7% vs. 13%, *p* = 0.002^a^	Organ/space SSI 5% vs. 9%, *p* = 0.06
Yamamoto M, et al.^8^	2020	Single‐center	Gastrointestinal or hernia surgery	Double usage of olanexidine	Total SSI	Incisional SSI 3.1% vs. 1.5%, *p* = 0.337
		Randomized controlled trial			3.1% vs. 2.0%, *p* = 0.537	Deep SSI *p* = 1.000
Kojima K, et al.^9^	2021	Single‐center	Colorectal cancer surgery	10% povidone‐iodine	SSI rate	Skin‐related side effects
		Propensity score matching analysis			8.6% vs. 8.6%, *p* = 1.000	13.8% vs. 0.0%
Fujita T, et al.^10^	2022	Single‐center	Thoracic esophagectomy	1.0% chlorhexidine	30‐days SSI	Superficial incisional SSI 0.8% vs. 0.8%, *p* = 0.990
		Propensity score matching analysis			12.0% vs. 20.1%, *p* = 0.090	Deep incisional SSI 1.7% vs. 3.5%, *p* = 0.390
						Organ/space SSI 10.3% vs. 15.7%, *p* = 0.300
Kubo N, et al.^11^	2022	Single‐center	Stomach and colon cancer surgery	10% povidone‐iodine	Total SSI	Superficial incisional SSI 2.2% vs. 8.6%, *p* = 0.034^a^
		Retrospective study			2.7% vs. 10.3%, *p* = 0.020^a^	Deep incisional SSI 0.5% vs. 1.7%, *p* = 0.371
This study	2022	Single‐center	Operative wound ≥2	10% povidone‐iodine	30‐day SSI	Superficial incisional SSI 8.1% vs. 9.5%, *p* = 0.544
		Randomized controlled trial	Gastrointestinal and hepatobiliary‐pancreatic surgery		12.8% vs. 18.0%, *p* = 0.083	Organ/space SSI 6.1% vs. 10.5%, *p* = 0.049^a^

*Note*: Values are the mean ± SD.

Abbreviation: SSI, surgical site infection.

^a^ Parameters with *p* < 0.05.

The Center for Disease Control guidelines recommend the use of alcohol‐containing disinfectants for surgical sites.^15^ This recommendation was established based on the previous randomized controlled trials on chlorhexidine reported by Darouiche et al. (10% vs. 16%, adjusted risk ratio: 0.59, 95% confidence interval: 0.41–0.85, *p* = 0.004) and Tuuli et al. (4% vs. 7%, adjusted risk ratio: 0.55, 95% confidence interval: 0.34–0.90, *p* = 0.020) relative to povidone‐iodine.^5^, ^6^ However, these previous studies have heterogeneity in the concentrations of both chlorhexidine (0.5%–4.0%) and povidone‐iodine (0.75%–10.0%), and the most suitable antisepsis has not been clearly established. Therefore, the results of the comparative studies of 1.5% olanexidine, including the present study, may provide a new interpretation for SSI prevention because the concentration of olanexidine in every study was the same, and 10% povidone‐iodine was used as a control arm in four of the five studies. In Japan, povidone‐iodine is mostly used as a preoperative antiseptic, not chlorhexidine, because chlorhexidine contains alcohol; therefore, it is flammable. The Pharmaceuticals and Medical Devices Agency, which is a Japanese regulatory agency that works together with the Ministry of Health, Labour, and Welfare, has strongly warned of the danger of the ignition of alcohol‐based antisepsis because of the use of electric scalpels.^7^ Because of this, we selected 10% povidone‐iodine as the reference arm for this clinical trial.

Several factors have been previously reported as risk factors for SSI incidence. The known risk factors for SSI include ASA‐PS, BMI, operating time, intraoperative blood transfusion, colorectal surgery, prevalence of diabetes, operative wound class, and nutritional status.^21^, ^22^, ^23^ In the present study, the SSI incidence in the 10% povidone‐iodine group was significantly higher in patients who underwent open surgery, with lower serum albumin, and with a prevalence of diabetes. The ultimate impact of new antisepsis is to reduce SSI in patients having risk factors for SSI as Obara et al. previously reported.^7^ However, 1.5% olanexidine could not close gaps of SSI incidences of patients with malnutrition (Alb <3.0 g/dL) and/or prevalence of diabetes. These results might be brought about by disproportion of timing of surgery and operative wound class.

Subgroup analyses revealed that emergent surgery and operative wound class III/IV did not significantly increase SSI incidence in 10% povidone‐iodine group. Emergent surgeries are often a contaminated or infected condition in clinical practice, however, it is controversial whether an emergent surgery is a risk factor of SSI incidence or not.^24^, ^25^, ^26^ We placed subcutaneous drain or performed closed incision negative pressure therapy when the operative wound was highly contaminated. These treatments might reduce the SSI incidence in patients with operative wound class III/IV.

Laparoscopic surgery has been widely accepted as a minimally invasive surgery because of its small incisions and lower blood loss compared with open surgery. Another advantage of laparoscopic surgery is a decreased SSI. Obara et al. reported that the laparoscopic approach significantly decreased SSI incidence in the 1.5% olanexidine group (4% vs. 12%, adjusted risk ratio: 0.35, 95% confidence interval: 0.17–0.74).^7^ In contrast, Kubo et al. could not demonstrate an additional effect of the laparoscopic approach in the 1.5% olanexidine group (effect size 0.636, 95% confidence interval: 0.138–2.930, *p* = 0.562).^11^ Our study also could not demonstrate the advantage of laparoscopic surgery (12.3% vs. 11.6%, adjusted risk ratio: 1.054, 95% confidence interval: 0.652–1.704); however, some reasons can explain this result: (1) We enrolled patients undergoing surgical procedures with intestinal resection. (2) We enrolled patients with operative wound class III and IV surgeries. (3) Some patients with perforated peritonitis and T4b colorectal cancers underwent laparoscopic resection. Therefore, the advantages of laparoscopic surgery might be neutralized. On the other hand, SSI incidence of open approach in the 1.5% olanexidine group was significantly lower compared to that in the 10% povidone‐iodine group. This may be explained by the long‐acting bactericidal effect of 1.5% olanexidine, however, there might be complicated biases increasing SSI incidence in patients undergoing open surgery in 10% povidone‐iodine group.^19^ Laparoscopic approach for perforated appendicitis significantly decreased the incisional SSI, however, organ/space SSI incidences were similar in both approaches.^27^ Conversely, we demonstrated a significant decrease of the organ/space SSI incidence in 1.5% olanexidine group. To clarify the bactericidal effects of olanexidine for contaminated or infected surgery, we suggest further powered, multicenter prospective clinical trials in the near future.

Theoretically, every antiseptic has a significant impact on superficial incisional SSI because infiltration of antiseptic is limited in skin. However, this study demonstrated that 1.5% olanexidine significantly reduced organ/space SSI. Some previous reports revealed that neoadjuvant chemotherapy, radiotherapy, preoperative parenteral nutrition, prolonged intensive care unit stay, disseminated cancer, preoperative sepsis, prevalence of inflammatory bowel syndrome, and bowel obstruction/perforation/fistula were identified as risk factors of organ/space SSI.^25^, ^26^ Furthermore, Gomila et al. and Xu et al. also clarified that stoma creation in colorectal surgery was the risk factor of organ/space SSI. Although our cohort included the largest number of patients undergoing lower gastrointestinal surgery, previously mentioned risk factors were not considered as adjustment factors.^28^, ^29^ Combinations of these factors might be confounding biases in 10% povidone‐iodine group.

In this study, we did not adopt timing of surgery and operative wound class as adjustment factors; therefore, allocation of the enrolled patients in 1.5% olanexidine groups was disproportionate to 10% povidone‐iodine group. This unequal enrollment might contradict the significant effect of 1.5% olanexidine. Subgroup analysis should be evaluated as underpowered analysis, however, every result of subgroup analysis revealed that 1.5% onalexidine has a potential of preventing SSI. Since the results of the subgroup analysis were only for reference, in the future, a trial limited to elective surgery and non‐contaminated wounds is warranted to verify the hypothesis.

Regarding the bactericidal spectrum of olanexidine, Seyama et al. evaluated the bactericidal effects of olanexidine by time–kill assays, demonstrating that Gram‐positive bacteria (*Enterococcus faecalis*, methicillin‐resistant *Staphylococcus aureus*, and so on) and Gram‐negative bacteria (*Acinetobacter baumannii*, *Enterobacter cloacae*, extended spectrum β‐lactamase‐producing *Klebsiella pneumoniae*, *Escherichia coli*, *Pseudomonas aeruginosa*, *Serratia marcescens*, *Bacteroides fragilis*, and so on) have been promptly killed within 1 min.^30^ Inoue et al. also compared the minimum bactericidal concentration of olanexidine, chlorhexidine, and povidone‐iodine against methicillin‐resistant *Staphylococcus aureus* and vancomycin‐resistant *Enterococcus* sp. and found that the minimum bactericidal concentration of olanexidine is equal to or lower than that of chlorhexidine and povidone‐iodine.^31^ Our study revealed that 1.5% olanexidine reduced the number of skin flora and SSI‐causing bacteria relative to 10% povidone‐iodine. Our study also demonstrated that 1.5% olanexidine reduced the concordance rate between SSI‐causing bacteria and wound‐cultured bacteria relative to 10% povidone‐iodine (*Enterococcus faecalis*: 66.7% vs. 87.5%, *Escherichia coli*: 0.0% vs. 100.0%). These results suggest that olanexidine has a substantive disinfectant effect on a wide range of bacteria during surgery and that it may be a strong alternative for preoperative antiseptics in preventing intraoperative bacterial colonization to the wound and bacterial growth in the skin.^17^

Our study has several limitations. First, the present study was a randomized prospective clinical trial conducted at a single center (Morioka Municipal Hospital). We enrolled several types of gastrointestinal surgery with intestinal resection at any time and for any operative wound class. This concept reflects actual clinical practice, not only in Japan but also in other countries. However, we could not adopt timing of surgery and operative wound class as adjustment factors because these factors were not always preoperatively determinate factors; therefore, there were preoperatively significant differences in these important factors. We did not enroll patients who underwent hepatectomy, splenectomy, or cholecystectomy because these procedures could be achieved without the exposure of intestinal contents. Regarding esophagectomy, we usually transfer patients with esophageal cancer to high‐volume centers. To resolve this limitation, various types of surgery, from common diseases to highly difficult surgery, should be included in the next study. Second, the full enrollment of the calculated sample size in both groups was completed in 2.5 years. The time span might affect the quality of the surgical procedure because of personnel changes and perioperative management of every disease through revised practical guidelines or concomitant ongoing studies. Third, clinical staging, the extent of lymph node dissection, preoperative chemotherapy, and adjacent organ resection were not considered as factors affecting SSI in patients with malignant diseases. Fourth, the present study was conducted as an investigator‐initiated clinical trial, and we performed all the processes of this trial. Furthermore, the colors of 1.5% olanexidine and 10% povidone‐iodine are clearly different; therefore, we could not conduct this study as a double‐blinded clinical trial. Finally, the current comparative studies on olanexidine were from Japan, and almost all enrolled patients in these studies were Japanese. Thus, the use of olanexidine should be expected to spread worldwide, and further studies of olanxidine are warranted to clarify its efficacy as a new antiseptic.

## 5 CONCLUSIONS

This randomized prospective clinical trial revealed that 1.5% olanexidine reduced the 30‐day overall SSI incidence relative to 10% povidone‐iodine during gastrointestinal surgery with intestinal resection; however, the result was not significant. Our study demonstrated that 1.5% olanexidine was associated with significantly reducing organ/space SSI and subgroup analyses also revealed that emergency surgery and operative wound class did not increase SSI incidence. Although the present study enrolled patients undergoing both emergency surgery and contaminated or infected surgery to reflect routine clinical practice, our results contribute to SSI management for patients who have undergone various gastrointestinal surgeries with any operative wound class and at any time. Further comparative studies may strengthen the evidence of olanexidine as a preoperative antiseptic agent.

## AUTHOR CONTRIBUTIONS

AU and TS contributed to conception and design, and drafted the article. AU, HF, KH, SA, NT, and YT acquired the data. AU and NT performed randomization and statistical analyses. AU, TS, HF, KH, SA, and NT managed the clinical treatments and investigation. AS and TS critically revised the article. All authors approved the final version of the manuscript to be submitted.

## FUNDING INFORMATION

This research did not receive any grant from funding agencies in the public, commercial, or not‐for‐profit sectors.

## CONFLICT OF INTEREST STATEMENT

The authors declare no competing interests.

## ETHICAL APPROVAL

Approval of the research protocol: This study was approved by the institutional ethics committee of Morioka Municipal Hospital (H30‐1) in accordance with the Declaration of Helsinki (1964) and its later amendments or comparable ethical standards.

Registry and the registration No. of the study/trial: This study was also registered to national clinical trials registry (UMIN000033830) prior to initiation of patient recruitment.

Informed consent: We obtained informed consent from each patient before enrollment, and patient anonymity was strictly protected.

Animal studies: This research was not an animal study.

## References

[1] Ban KA , Minei JP , Laronga C , Harbrecht BG , Jensen EH , Fry DE , et al. American College of Surgeons and surgical infection society: surgical site infection guidelines, 2016 update. J Am Coll Surg. 2017;224:59–74. 27915053 10.1016/j.jamcollsurg.2016.10.029

[2] Ohge H , Mayumi T , Haji S , Kitagawa Y , Kobayashi M , Kobayashi M , et al. The Japan Society for Surgical Infection: guidelines for the prevention, detection, and management of gastroenterological surgical site infection, 2018. Surg Today. 2021;51:1–31. 33320283 10.1007/s00595-020-02181-6 PMC7788056

[3] De Simone B , Sartelli M , Coccolini F , Ball CG , Brambillasca P , Chiarugi M , et al. Intraoperative surgical site infection control and prevention: a position paper and future addendum to WSES intra‐abdominal infections guidelines. World J Emerg Surg. 2020;15:10. 32041636 10.1186/s13017-020-0288-4 PMC7158095

[4] Japan Nosocomial Infections Surveillance . Ministry of Health, Labour and Welfare. Accessed August 25, 2022. https://janis.mhlw.go.jp/report/ssi.html

[5] Darouiche RO , Wall MJ Jr , Itani KM , Otterson MF , Webb AL , Carrick MM , et al. Chlorhexidine‐alcohol versus povidone‐iodine for surgical‐site antisepsis. N Engl J Med. 2010;362:18–26. 20054046 10.1056/NEJMoa0810988

[6] Tuuli MG , Liu J , Stout MJ , Martin S , Cahill AG , Odibo AO , et al. A randomized trial comparing skin antiseptic agents at cesarean delivery. N Engl J Med. 2016;374:647–55. 26844840 10.1056/NEJMoa1511048 PMC4777327

[7] Obara H , Takeuchi M , Kawakubo H , Shinoda M , Okabayashi K , Hayashi K , et al. Aqueous olanexidine versus aqueous povidone‐iodine for surgical skin antisepsis on the incidence of surgical site infections after clean‐contaminated surgery: a multicentre, prospective, blinded‐endpoint, randomised controlled trial. Lancet Infect Dis. 2020;20:1281–9. 32553191 10.1016/S1473-3099(20)30225-5

[8] Yamamoto M , Hara K , Sugezawa K , Uejima C , Tanio A , Tada Y , et al. Disinfection with single or double usage of new antiseptic olanexidine gluconate in general surgery: a randomized study. Langenbecks Arch Surg. 2020;405:1183–9. 33057823 10.1007/s00423-020-02007-6

[9] Kojima K , Nakamura T , Habiro T , Waraya M , Hayashi K , Ishii KI . Examination of the efficacy of olanexidine gluconate for surgical site infections in colorectal cancer elective surgery. J Infect Chemother. 2021;27:1729–34. 34521590 10.1016/j.jiac.2021.08.019

[10] Fujita T , Okada N , Sato T , Sato K , Fujiwara H , Kojima T , et al. Propensity‐matched analysis of the efficacy of olanexidine gluconate versus chlorhexidine‐alcohol as an antiseptic agent in thoracic esophagectomy. BMC Surg. 2022;22:20. 35065644 10.1186/s12893-022-01480-8 PMC8783436

[11] Kubo N , Furusawa N , Takeuchi D , Imai S , Masuo H , Umemura K , et al. Clinical study of a new skin antiseptic olanexidine gluconate in gastrointestinal cancer surgery. BMC Surg. 2022;22:194. 35590405 10.1186/s12893-022-01641-9 PMC9118739

[12] Schulz KF , Altman DG , Moher D , CONSORT Group . CONSORT 2010 statement: updated guidelines for reporting parallel group randomised trials. BMJ. 2010;(340):c332. 20332509 10.1136/bmj.c332 PMC2844940

[13] Bhangu A , Singh P , Lundy J , Bowley DM . Systemic review and meta‐analysis of randomized clinical trials comparing primary vs delayed primary skin closure in contaminated and dirty abdominal incisions. JAMA Surg. 2013;148:779–86. 23803860 10.1001/jamasurg.2013.2336

[14] Duttaroy DD , Jitendra J , Duttaroy B , Bansal U , Dhameja P , Patel G , et al. Management strategy for dirty abdominal incisions: primary or delayed primary closure? A randomized trial. Surg Infect (Larchmt). 2009;10:129–36. 19388835 10.1089/sur.2007.030

[15] Berríos‐Torres SI , Umscheid CA , Bratzler DW , Leas B , Stone EC , Kelz RR , et al. Centers for Disease Control and Prevention guideline for the prevention of surgical site infection, 2017. JAMA Surg. 2017;152:784–91. 28467526 10.1001/jamasurg.2017.0904

[16] Mayhew D , Mendonca V , Murthy BVS . A review of ASA physical status—historical perspectives and modern developments. Anaesthesia. 2019;74:373–9. 30648259 10.1111/anae.14569

[17] Hagi A , Iwata K , Nii T , Nakata H , Tsubotani Y , Inoue Y . Bactericidal effects and mechanism of action of Olanexidine gluconate, a new antiseptic. Antimicrob Agents Chemother. 2015;59:4551–9. 25987609 10.1128/AAC.05048-14 PMC4505255

[18] Nii T , Yumoto H , Hirota K , Miyake Y . Anti‐inflammatory effects of olanexidine gluconate on oral epithelial cells. BMC Oral Health. 2019;19:239. 31703580 10.1186/s12903-019-0932-0 PMC6839112

[19] Shinzato Y , Sakihara E , Kishihara Y , Kashiura M , Yasuda H , Moriya T . Clinical application of skin antisepsis using aqueous olanexidine: a scoping review. Acute Med Surg. 2022;9:e723. 35028157 10.1002/ams2.723 PMC8741875

[20] Asukai M , Ohishi T , Fujita T , Suzuki D , Nishida T , Sugiura K , et al. Olanexidine gluconate versus povidone‐iodine for preventing surgical‐site infection in orthopaedic surgery: a retrospective study. J Orthop Sci. 2019;24:1125–9. 31395421 10.1016/j.jos.2019.07.008

[21] Cheadle WG . Risk factors for surgical site infection. Surg Infect (Larchmt). 2006;7:S7–11. 16834549 10.1089/sur.2006.7.s1-7

[22] Ejaz A , Schmidt C , Johnston FM , Frank SM , Pawlik TM . Risk factors and prediction model for inpatient surgical site infection after major abdominal surgery. J Surg Res. 2017;217:153–9. 28595819 10.1016/j.jss.2017.05.018

[23] Kulkarni N , Arulampalam T . Laparoscopic surgery reduces the incidence of surgical site infections compared to the open approach for colorectal procedures: a meta‐analysis. Tech Coloproctol. 2020;24:1017–24. 32648141 10.1007/s10151-020-02293-8 PMC7346580

[24] Gillespie BM , Harbeck E , Rattray M , Liang R , Walker R , Latimer S , et al. Worldwide incidence of surgical site infections in general surgical patients: a systematic review and meta‐analysis of 488,594 patients. Int J Surg. 2021;95:106136. 34655800 10.1016/j.ijsu.2021.106136

[25] Lawson EH , Hall BL , Ko CY . Risk factors for superficial vs deep/organ‐space surgical site infections: implications for quality improvement initiatives. JAMA Surg. 2013;148:849–58. 23864108 10.1001/jamasurg.2013.2925

[26] Sun C , Gao H , Zhang Y , Pei L , Huang Y . Risk stratification for organ/space surgical site infection in advanced digestive system cancer. Front Oncol. 2021;11:705335. 34858805 10.3389/fonc.2021.705335 PMC8630667

[27] Galli R , Banz V , Fenner H , Metzger J . Laparoscopic approach in perforated appendicitis: increased incidence of surgical site infection? Surg Endosc. 2013;27:2928–33. 23443482 10.1007/s00464-013-2858-y

[28] Gomila A , Carratalà J , Camprubí D , Shaw E , Badia JM , Cruz A , et al. Risk factors and outcomes of organ‐space surgical site infections after elective colon and rectal surgery. Antimicrob Resist Infect Control. 2017;6:40. 28439408 10.1186/s13756-017-0198-8 PMC5401556

[29] Xu Z , Qu H , Gong Z , Kanani G , Zhang F , Ren Y , et al. Risk factors for surgical site infection in patients undergoing colorectal surgery: a meta‐analysis of observational studies. PLoS One. 2021;16:e0259107. 34710197 10.1371/journal.pone.0259107 PMC8553052

[30] Seyama S , Nishioka H , Nakaminami H , Nakase K , Wajima T , Hagi A , et al. Evaluation of in vitro bactericidal activity of 1.5% Olanexidine gluconate, a novel Biguanide antiseptic agent. Biol Pharm Bull. 2019;42:512–5. 30568106 10.1248/bpb.b18-00821

[31] Inoue Y , Hagi A , Nii T , Tsubotani Y , Nakata H , Iwata K . Novel antiseptic compound OPB‐2045G shows potent bactericidal activity against methicillin‐resistant Staphylococcus aureus and vancomycin‐resistant enterococcus both in vitro and in vivo: a pilot study in animals. J Med Microbiol. 2015;64:32–6. 25351713 10.1099/jmm.0.080861-0

